# Stone/Coating Interaction and Durability of Si-Based Photocatalytic Nanocomposites Applied to Porous Lithotypes

**DOI:** 10.3390/ma11112289

**Published:** 2018-11-15

**Authors:** Marco Roveri, Francesca Gherardi, Luigi Brambilla, Chiara Castiglioni, Lucia Toniolo

**Affiliations:** 1 Politecnico di Milano, Dipartimento di Chimica, Materiali e Ingegneria Chimica “G. Natta”, 20133 Milano, Italy; luigi.brambilla@polimi.it (L.B.); chiara.castiglioni@polimi.it (C.C.); lucia.toniolo@polimi.it (L.T.); 2 School of Chemistry, University of Lincoln, LN6 7DL Lincoln, UK; fgherardi@lincoln.ac.uk

**Keywords:** TiO_2_ nanoparticles, alkylalkoxysilane, stone protection, water-repellency, photocatalysis, UV ageing, artificial rain, photo-oxidative degradation, durability

## Abstract

The use of hybrid nanocoatings for the protection of natural stones has received increasing attention over the last years. However, the interaction of these materials with stones and, in particular, its modification resulting from the blending of nanoparticles and matrices, are yet little explored. In this work, the interaction of two nanocomposite coatings (based on alkylalkoxysilane matrices and TiO_2_ nanoparticles in water and 2-propanol) with two different porous stones is examined in detail by comparing their absorption behaviour and protection performance with those of the respective TiO_2_-free matrices. It is shown that the protective effectiveness of both matrices is not negatively affected by the presence of TiO_2_, as the desired water barrier effect is retained, while a significant photocatalytic activity is achieved. The addition of titania leads to a partial aggregation of the water-based matrix and accordingly reduces the product penetration into stones. On the positive side, a chemical interaction between titania and this matrix is observed, probably resulting in a greater stability of nanoparticles inside the protective coating. Moreover, although an effect of TiO_2_ on the chemical stability of matrices is observed upon UV light exposure, the protective performance of coatings is substantially maintained after ageing, while the interaction between matrices and nanoparticles results in a good retention of the latter upon in-lab simulated rain wash-out.

## 1. Introduction

The use of TiO_2_ nanoparticles as protective treatments for natural stones has aroused much interest over the last 10 years [1,2,3,4,5], prompting efforts to enhance their effectiveness as photocatalytic agents in this specific field of application. Indeed, one of the main drawbacks of using nanoTiO_2_ dispersions is that they have poor adhesion to the stone substrates, thus tending to penetrate into the pore structure or be easily removed by the mechanical action of wind and rainfall. In both cases, this results in a significant decrease in the titania surface content and thus in lower photocatalytic performances [6,7]. One general strategy towards overcoming this issue has been represented by the development of TiO_2_-based nanocomposites, which make up a wide class of hybrid materials obtained from the addition of titania nanoparticles to organo-modified silica precursors such as TEOS/alkylalkoxysilanes or different siloxane/acrylic/fluorinated/epoxy polymer dispersions [8,9,10,11,12,13,14,15,16,17,18,19,20,21,22]. These composite materials have displayed a number of advantages over bare nanoTiO_2_ dispersions, which include a better adhesion to the stone substrates, a lower tendency towards unwanted nanoparticle aggregation and the ability to combine a self-cleaning photocatalytic action with the properties of traditional water-repellent or consolidation treatments [20,21,22], while retaining a good aesthetic compatibility. Indeed, a strict requirement in this kind of application is that coatings should not change the “appearance” of stones, that is, their surface colour, texture and finishing.

From the viewpoint of materials characterization, the properties of some of these hybrid coatings have been very well described: in particular, investigations have focused on elucidating the structure of the titania-polymer interface, pointing out the stabilizing effect of filler-matrix interactions and the modification of the wetting behaviour of the composites due to the rough surface topography induced by TiO_2_ nanoparticles [23,24]. Furthermore, as regards the application on stone, the protective performance of several TiO_2_-based nanocomposites has been assessed on different stone substrates, including both high and low porosity stones [11,13,22]. Specifically, the contribution of nanoparticles to the reduction of surface wettability has been shown on low porosity stones such as marbles [19,21], while the photocatalytic performance and self-cleaning behaviour of nanoTiO_2_ and the influence of different polymer matrices have been widely investigated on both high and low porosity stones [11,19,22].

However, few studies did investigate in more detail how the combination of nanoparticles and polymer matrices affects the properties of the coatings in their interaction with stone substrates [10,11,19]. Furthermore, there is still very limited information on the durability of these coatings under real outdoor conditions [21,25,26]. Accelerated ageing procedures have indeed been performed to assess the effects of rain wash-out [27,28,29,30], wet-dry cycles [31], exposure to UV light [26,27,29,30,31,32] and soiling [27,30], especially with regard to the adhesion of titania nanoparticles to the stone substrates and the evaluation of the change in their photocatalytic activity. However, the results obtained on different stones and cementitious materials are far from univocal and it has been suggested that the intrinsic properties of stones play a relevant role in the long-term efficiency of the products and result in different durability issues [25,26,33]. Furthermore, only few studies have set out to address the influence of photo-active TiO_2_ nanoparticles on the durability of hybrid coatings upon solar light irradiation [20,34] and, in particular, to clarify the effects on their hydrophobic properties due to the inherent UV-induced superhydrophilicity of titania and to the possible photo-catalysed oxidative degradation of matrices.

In this work, which is part of a wider-scope research dealing with the set-up and testing of innovative nanocomposite materials for the conservation of architectural heritage, the study of the interaction of two different TiO_2_-based nanocomposite formulations with two porous stones of high relevance in the built heritage field is addressed. These formulations, consisting of alkylalkoxysilane reactive sols combined with different titania nanoparticles, were developed in the framework of the EU-funded NanoCathedral project [35] and their performance has already been discussed in a previous study [36]. In the present research, the interaction of these nanocomposites with stones and their protective performance are compared to those of the corresponding alkylalkoxysilane matrices, especially in terms of absorption behaviour, surface textural modification and achieved water-repellency, focusing on the role played therein by the combination of TiO_2_ nanoparticles with matrices. Then, an investigation of the durability of nanocomposites and matrices is carried out in order to assess the contribution of TiO_2_ to the possible photo-oxidative degradation of nanocomposites upon UV light exposure and check the retention of nanoparticles on the stone surfaces upon rain wash-out.

## 2. Materials and Methods

**Figure 1:**
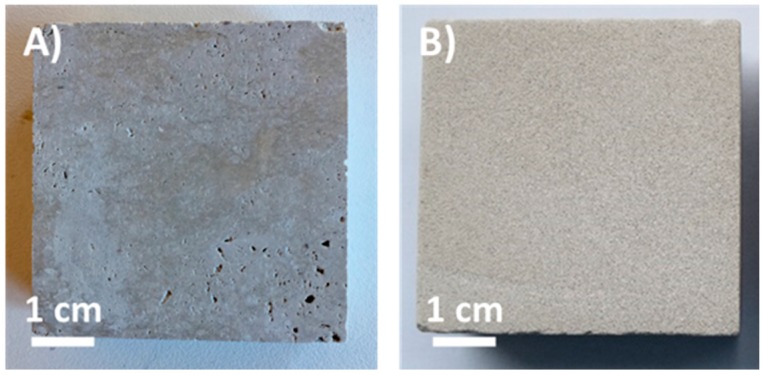
Photographs of Ajarte (**A**) and Obernkirchen (**B**) stone specimens.

Two different high porosity stones (Figure 1), characterized by a calcareous or siliceous composition, were used. Ajarte (Lumaquela de Ajarte) is a sedimentary rock from Treviño area (Spain), having a calcite matrix with a high amount of intercrystalline pores. Obernkirchen is a quartzarenite from Bückeberge area (Germany), characterized by a fine-grained texture. These natural stones have particular relevance in the cultural heritage field: Ajarte limestone was widely employed as building material in the north of Spain since the Middle Ages and is found in monuments such as St. Mary’s Cathedral in Vitoria-Gasteiz (XIII-XVI cent.), while Obernkirchen sandstone is especially renowned for being one of the materials used in the construction of Cologne Cathedral (XIII-XIX cent.).

The two nanocomposite formulations used in this study, hereafter referred to as WNC and ANC, were developed in the framework of the EU-funded H2020 NanoCathedral project (Grant Agreement n. 646178) by two SME Partners. They consist of TiO_2_ nanoparticles (Colorobbia Consulting srl, Sovigliana Vinci, Italy) dispersed in commercial alkylalkoxysilane reactive sols (ChemSpec srl, Peschiera Borromeo, Italy). The details of the preparation, including the commercial names of product components, are protected by a non-disclosure agreement. The main properties of these components are shown in Table 1, while the composition of the formulates is reported in Table 2.

Some of the properties of WNC and ANC have been assessed in previous studies [36,37]. The former is a water-based dispersion of alkylalkoxysilane *oligomers* (15% *w*/*w*) and TiO_2_ nanoparticles (0.96% *w*/*w*), while the latter is an alcohol-based solution of alkylalkoxysilane *monomers* (40% *w*/*w*) with a lower titania concentration (0.12% *w*/*w*). The corresponding TiO_2_-free alkylalkoxysilane matrices are hereafter referred to as m-WNC and m-ANC, respectively.

Rheological measurements on the nanocomposites and corresponding matrices were carried out through a Bohlin CV0 120 Rheometer (Bohlin Instruments Vertriebs GmbH, Pforzheim, Germany), using a cone-plate configuration (1° angle, 40 mm diameter) with 0.03 mm gap. Flow curves were recorded for 3 min under 0.1 to 3 Pa stress at 20 °C. Since the fluids exhibited a shear-thinning behaviour, the value of viscosity measured in the low shear rate region around 10 s^−1^ was assumed to be representative of the rheological behaviour of the products in a capillary flow regime. Two measurements were performed for each product. Particle size was measured on a 90 Plus Dynamic Laser Light Scattering instrument (Brookhaven Instruments Corporation, Holtsville, NY, USA) equipped with a 35-mW Laser and an Avalanche photodiode detector collecting the scattered light at 90°. Three measurements were performed for each product.

In order to assess the crystalline form of TiO_2_ nanoparticles and to investigate their interaction with alkylalkoxysilane matrices upon curing, Raman spectra of TiO_2_ dispersions (nTiO_2_-W and nTiO_2_-A, the latter being previously diluted in 2-propanol), alkylalkoxysilane matrices (m-WNC and m-ANC) and different combinations of them (TiO_2_: alkylalkoxysilane in 10:1, 2:1, 1:1, 1:5 *w*/*w* ratio) were recorded after keeping the sols in open vials under controlled humidity and temperature (50% RH and 23 °C) for 40 days until solvent evaporation and curing. Raman spectra of these samples were recorded using a Horiba Jobin Yvon Labram HR800 Raman spectrometer coupled with an Olympus BX41 microscope. The 514.5 nm excitation laser line (Ar^+^ Sabilite 2017 Spectra-Physics) at 2 mW power was focused by a 50X objective directly on samples deposited on a glass slide. Spectra were recorded by collecting 4 scans and integrating over 10 s. A baseline correction and a smoothing of signal were performed through OMNIC software (Thermo Fischer Scientific, Waltham, MA, USA).

Freshly quarried specimens (5 × 5 × 1 cm^3^ and 5 × 5 × 2 cm^3^ prisms, 2 and 3 for each size respectively) of Ajarte and Obernkirchen stones were gently polished with abrasive paper (P180 carborundum paper), washed and kept in deionized water for 1 h in order to remove any excess soluble salts. Afterwards, they were dried in oven at 65 °C until constant weight and stored in a silica gel desiccator for 24 h. The products and their respective matrices were applied by capillary absorption for 6 h, using a filter paper pad saturated with the treatments, according to EN standard [38]. After the application, the stone specimens were kept sheltered from direct light for 30 days at the temperature and humidity conditions of the lab (about 23 °C and 50% RH) in order to allow solvent evaporation and the curing of matrices. In order to determine the amount of absorbed product, stone specimens were weighed before and after treatment. The weights were divided by the respective product densities and by the areas of treated surfaces, yielding the volumes of liquid absorbed per unit area (μL/cm^2^). Both products and matrices were also cast on 2 glass slides (25 × 75 mm) that had been previously treated with hot Piranha solution (conc. H_2_SO_4_ and 30% *w*/*w* H_2_O_2_ in 3:1 volume ratio) for 15 min in order to increase the amount of reactive silanol groups on the glass surface. The slides were kept under saturated solvent (water/2-propanol) atmosphere until complete evaporation of the solvent and formation of a thin film and then stored in a closed vessel for 1 month in order to allow proper curing of the alkylalkoxysilane matrices.

The aesthetic compatibility of treatments was assessed through diffuse reflectance Vis-light spectroscopy (Konica Minolta CM-600D Vis spectrophotometer with a D65 illuminant at 8°, 360–740 nm wavelength range). 25 measurements were performed on each stone specimen before and after the application of treatments according to the EN standard protocol [39]. The results were expressed in the CIE L*a*b* colour space and the average values of L*, a* and b* were used to calculate the colour change ΔE* according to the formula ΔE* = [(L^*^_t_ − L^*^_nt_)^2^ + (a^*^_t_ − a^*^_nt_)^2^ + (b^*^_t_ − b^*^_nt_)^2^]^1/2^, where the subscripts t and nt stand for treated and untreated specimen, respectively. ΔE* values should not exceed the threshold value of 5 in order for a product to meet the aesthetic requirements for application in the cultural heritage field [40].

The surface morphology of stone specimens was characterized through Environmental Scanning Electron Microscopy (Zeiss EVO 50 EP) before and after the application of treatments. For the Atomic Force Microscopy (AFM) analysis of surface roughness, a Solver Pro AFM microscope (NT-MDT Spectrum Instruments, Beijing, China) was employed, using a silicon cantilever with a tip of 14–16 μm height (NSG10, NT-MDT) and tip curvature radius of 10 nm at a resonant frequency of 140–390 KHz. Measurements were performed in tapping mode at 0.6 Hz scan rate, with 2 scans of a 0.5 × 0.5 µm^2^ area. The acquired images were elaborated through the Nova SPM software (NT-MDT Spectrum Instruments). 4 specimens per lithotype were analysed before and after treatment (one specimen for each treatment).

Capillary water absorption was measured on untreated specimens and then after 1 and 2 months from the application of protective treatments according to EN standard [41]. All reported data refer to this latter set of measurements. The specimens were weighed at the following time intervals: 10 min, 20 min, 30 min, 60 min, 4 h, 6 h, 24 h, 48 h, 72 h and 96 h. The area under the absorption curve was calculated through numerical integration. The Relative Capillary Index (CI_rel_) was used to evaluate the behaviour of treated specimens for the duration of the experiment (96 h), while the Sorptivity (AC) was used to evaluate their short-term behaviour (30 min). For both parameters, values lower than 0.2 indicate a good reduction of water absorption [40]. Static contact angle (θ) measurements were performed on 15 points for each specimen, according to EN standard [42], using an OCA (Optical Contact Angle) 20 PLUS (DataPhysics, San Jose, CA, USA). A drop volume of 5 µL was used and measurements were performed 10 s after drop deposition. Drop profiles were analysed according to Laplace-Young theory. HPLC grade water (Chromasolv ^®^ Plus, Sigma Aldrich, St. Louis, MO, USA) was used as the liquid.

The photocatalytic properties of the products (WNC and ANC) were assessed through the Rhodamine discolouration test, according to the procedure reported in a previous work [36]. For a comparison between products and their respective TiO_2_-free matrices, the latters’ behaviour was tested as well. The irradiation chamber (Suntest XLS+, URAI S.p.A) was equipped with a Xenon arc lamp (NXE 1700, with a cut-off filter for λ < 295 nm) producing an irradiance of 765 W/m^2^ in the 300–800 nm range. The degradation of Rhodamine B was monitored up to 150 min by means of colour measurements. For the assessment of photocatalytic activity, the a* value from each measurement was considered, which represents the red colour component in the CIE Lab colour space. The extent of discolouration (D*) was then evaluated according to the formula D*(%) = (|a*(t) − a*(rB)|/|a*(rB) − a*(0)|)*100, where a*(0) and a*(rB) are the average values of chromatic coordinate a* before and after the application of the colourant solution and a*(t) is the a* value after t minutes of light exposure. Specimens treated with a commercial water-repellent product, Silres BS 290 (Wacker Chemie GmbH, Munich, Germany), based on a mixture of silanes and siloxanes (8% *w*/*w* in white spirit), were used as reference owing to their hydrophobic features making the interaction of the colourant solution with stones comparable with that observed on treated specimens. In order to distinguish the effects of photolytic and thermal degradation of Rhodamine from the actual photocatalytic process, the ratios of D* values for specimens treated with WNC, ANC and their respective matrices (D*_PRODUCT_) and for specimens treated with Silres (D*_SILRES_) at 30, 90 and 150 min are reported as parameters for the evaluation of photocatalytic activity. In the case of TiO_2_-free matrices, this ratio is clearly expected to approach unity, while for photocatalytic products greater than 1 values should be obtained.

Specimens treated with WNC, ANC, m-WNC and m-ANC (16 specimens per lithotype, including subsets of 5 × 5 × 2 cm^3^ and 5 × 5 × 1 cm^3^ specimens, previously characterized according to the testing protocol described above) were subjected to a UV ageing procedure in order to assess the chemical stability of the two organosilica gels and investigating the influence of TiO_2_ nanoparticles on possible photo-oxidative effects. Products and matrices applied on glass slides also underwent this ageing procedure. The UV ageing was conducted for 600 h in an irradiation chamber (Suntest XLS+, Atlas GmbH, Ganderkesee, Germany) equipped with a Xenon arc lamp (NXE 1700) simulating daylight (cut-off filter for λ < 295 nm). The irradiance of the lamp was set to 765 W/m^2^ in the 300–800 nm range, with an emission of about 65 W/m^2^ from 300–400 nm (as reported by the manufacturer), which is close to the hypothetical upper limit of UV irradiation of natural daylight (70 W/m^2^) [27]. The temperature of the specimens, measured through a black body reference, was kept at 65 ± 15 °C. This experimental set-up was comparable to others adopted in literature [27,29]. Water absorption and static contact angle measurements were then performed on aged specimens in order to assess the retention of protective effectiveness. The nanocomposites and corresponding matrices cast on glass slides were analysed through FTIR microscopy before and after ageing in order to characterize their chemical modification. μ-FTIR analysis was conducted in attenuated total reflection (ATR) mode (Ge crystal) on a Thermo Nicolet 6700 spectrophotometer coupled to a Thermo Nicolet Continuum FTIR microscope with MCT detector (128 acquisitions, 650–4000 cm^−1^ spectral window, 4 cm^−1^ resolution). Spectra were processed on OMNIC software (Thermo Fischer Scientific): the baseline was corrected and a reduction of noise was performed via the smoothing function. Secondly, for WNC and ANC, 2 further specimens per lithotype were subjected to a rain ageing procedure with the aim of assessing the mechanic stability of TiO_2_ nanoparticles under the action of rain wash-out. The resistance to rain wash-out was assessed by a purposely designed rain chamber, equipped with a peristaltic pump (Behr GmbH, New York, NY, USA) and a set of needles (d = 0.2 mm) providing constant dripping of distilled water with a rate of about 82 mm/h onto specimens placed on a rack and tilted by 45° with respect to the horizontal plane. The experimental set-up was in partial agreement with similar experiments reported in literature [28]. The test was conducted in 4 steps of 24 h, each step consisting of 7 h of wetting followed by 17 h of drying at room temperature. Each specimen was subjected to four rain drops and periodically displaced by 0.5 cm along the horizontal plane in order to obtain a more homogeneous distribution of the rain drops over the tested surface. To the same purpose, the specimens were also rotated by 180° after half testing time. Then, in order to assess the retention of TiO_2_ nanoparticles on the stone surfaces, the photocatalytic activity was measured again through the Rhodamine discolouration test.

## 3. Results and Discussion

### 3.1. Characterization of Materials

#### 3.1.1. Lithotypes

**Figure 2:**
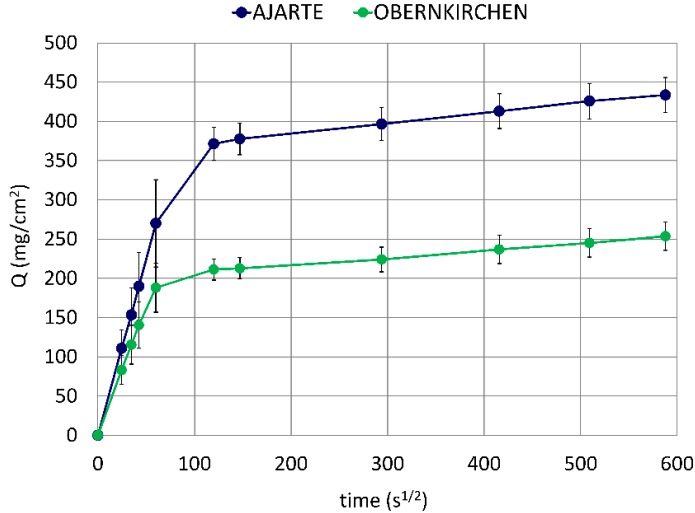
Water absorption by capillarity in untreated lithotypes.

Ajarte and Obernkirchen are highly porous stones (23.5 and 24.1 vol%, respectively) with different mineralogical and microstructural properties [36,37,43]. (These latter properties are recalled in Table S1 in Supplementary Materials). Ajarte has an almost purely calcitic composition (93.4 mol%) and a low average pore diameter (0.17 μm), whereas Obernkirchen consists mainly of quartz (89.6 mol%) and displays a considerably higher mean pore size (0.76 μm). Although both stones exhibit very close values of open porosity, the suction power and velocity of their capillary networks are remarkably different: Ajarte absorbs water to a far greater extent and at a faster rate than Obernkirchen (Figure 2), which is probably due to differences in pore shape and connectivity.

These distinct water absorption regimes will be taken into account when evaluating the penetration of protective treatments into the porous crystalline matrix of the two stones. Furthermore, the different pore-size distributions (see Figure S1 in Supplementary Materials), especially the greater amount of small-size pores found in Ajarte, will be considered as a possible discriminating factor for the behaviour of the products under study, as the latter need to penetrate into the widest possible pore-size range in order to produce a diffuse and effective water-repellent action.

#### 3.1.2. Protective Treatments

The two nanocomposite formulations WNC and ANC display hybrid properties. Upon curing of the alkylalkoxysilane matrices, alkyl groups impart hydrophobic features, while TiO_2_ nanoparticles at low concentration are able to provide photocatalytic and self-cleaning properties [36]. The sol-gel condensation of silane precursors upon solvent evaporation and curing produces, to a variable extent, a cross-linked organosilica gel network, in accordance with the usual reactivity of alkylalkoxysilane compounds. Silanol groups in the gels are expected to interact with stone substrates either through condensation with surface silanols of silicate minerals [44] or through the build-up of noncovalent interactions (hydrogen bonding). Furthermore, protonated amine groups (present in WNC) should be involved in ionic interactions with both carbonate and silicate minerals, as it is reported for other aminosilane compounds [45].

Since transport properties have a predictable effect on the penetration of treatments in porous substrates, viscosity and particle size measurements, previously performed on WNC and ANC [37], were also conducted on the corresponding matrices m-WNC and m-ANC in order to assess the modification of these properties upon addition of TiO_2_ nanoparticles. The results (Table 3) show that only quite small differences exist in the viscosity of fluids (WNC/m-WNC and ANC/m-ANC), whilst marked differences can be observed among the values of particle size. Actually, it is noteworthy that both m-WNC (which is made up of alkylalkoxysilane oligomers) and the respective TiO_2_ nanoparticles (nTiO_2_-W) consist of aggregates of several tens of nanometres (Table 1 and Table 3). Moreover, further aggregation of these silane oligomers and/or TiO_2_ nanoparticles probably occurs after the preparation of the mixture, because the final product turns out to have higher particle size compared to its separate components (Table 3). This suggests that the sol increases its aggregation state after the mixing of components and a greater likelihood exists that it will interact with the inner pore surface of stones during the capillary uptake, thus experiencing a more difficult penetration. Conversely, the matrix m-ANC consists of well dispersed alkylalkoxysilane monomers with no light scattering features (Table 3) and its corresponding nanotitania (nTiO_2_-A) exhibits a low particle size (Table 1). Furthermore, both components do not seem to undergo any aggregation upon mixing, since particles in the formulate (Table 3) are nearly the same size as those of the precursor TiO_2_ dispersion. Therefore, given the stability of the sol, which is typical of alcohol-based alkylalkoxysilanes, the product behaves as a non-reactive fluid during the absorption into stones, thus facilitating its own penetration. 

In conclusion, the significantly different particle size and distinct stability of the two nanocomposites towards aggregation are the discriminating factors for their penetration into porous substrates, more so in the case of Ajarte stone whose average pore diameter is around 200 nm (Table S1). In particular, the greater size of WNC particles, which exceeds 100 nm, as well as the lower stability of its components (as it is the case of water-based alkylalkoxysilanes, characterized by a higher reactivity as compared to their solvent-based analogues [46]) can be expected to prevent the penetration of the sol into the thinnest pores and are likely to reduce it for the whole of the other pores. On the other hand, the lower size of TiO_2_ nanoparticles in ANC and the stability of its sol components should concur to a greater and more homogeneous penetration of the product into a wider pore size range. From this viewpoint, little difference is to be expected between ANC and its TiO_2_-free matrix m-ANC, whereas WNC should penetrate less easily than m-WNC.

**Figure 3:**
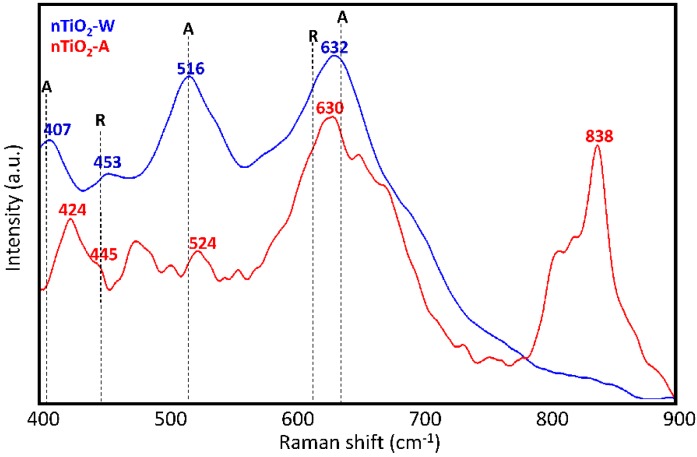
Raman spectra of dry TiO_2_ dispersions (nTiO_2_-W and n-TiO_2_-A) with indication of peak positions for anatase (A) and rutile (R) crystals.

Raman analysis of the TiO_2_ dispersions (nTiO_2_-W and nTiO_2_-A) used to prepare the two formulations was performed after solvent evaporation with the aim of assessing the titania polymorphs present therein. As it is well known, among these polymorphs, pure anatase or anatase in the presence of a small fraction of rutile display the highest photocatalytic activity [47,48]. Raman spectra in Figure 3 reveal that the pattern of nTiO_2_-W (632, 516 and 407 cm^−1^), that is, TiO_2_ nanoparticles used in WNC formulation, bears a rather good correspondence to that of anatase [49], in spite of peaks at 407 cm^−1^ and 632 cm^−1^ being displaced by about 8 cm^−1^ (upward and downward, respectively) relative to the corresponding signals of the anatase crystal. The presence of some residual rutile phase can also be argued on the basis of the weak signal at 453 cm^−1^ [49]. The observed broadening and peak shift of the Raman signals can be possibly ascribed both to the non-stoichiometry of the samples (i.e., an oxygen deficiency) and to the presence of disorder in TiO_2_ nanoaggregates [50]. Moreover, it is well known that confinement effects in nanosized crystalline domains is responsible of a partial relaxation of the Raman selection rules for the crystal, with consequent activation of phonons with wave-vector close to the Γ point in the first Brillouin zone. The above phenomenon is often reported as the main responsible for band broadening, changes of the band shapes and displacements of the band maxima observed in the vibrational spectra of nanocrystals. In the case of nTiO_2_-A, that is, TiO_2_ nanoparticles used in ANC formulation, the interpretation of spectral features turns out to be more complicated. Except for the intense band at 630 cm^−1^, suggesting the presence of anatase (with the same shift observed for nTiO_2_-W) and a weaker signal at 524 cm^−1^ that could be given the same interpretation, the third expected signal around 399 cm^−1^ might be identified as the feature observed at 424 cm^−1^. Furthermore, other signals, not ascribable to either anatase or rutile phase, can be noticed. In particular, the intense band at 838 cm^−1^, which is due to 1,2-propanediol, indicates that residual solvent is still present in the solid phase, probably adsorbed or covalently bonded [51] to the surface of TiO_2_ nanoparticles.

**Figure 4:**
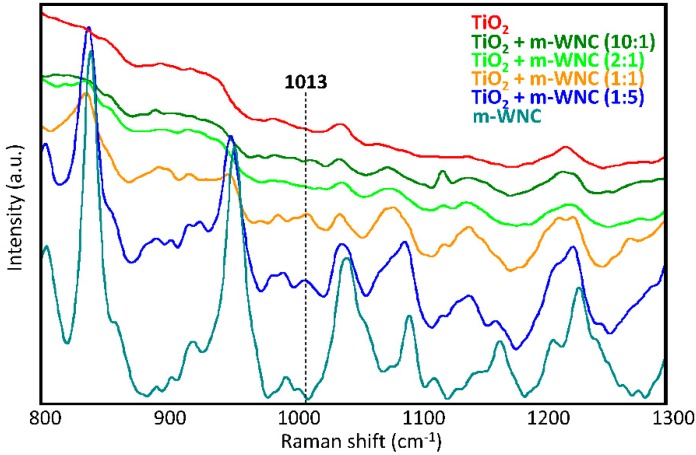
Raman spectra of dry nTiO_2_-W/m-WNC mixtures at different weight ratios: pure TiO_2_, 10:1, 2:1, 1:1, 1:5 and pure m-WNC.

In order to assess whether a modification in the chemical environment around TiO_2_ nanoparticles occurs by interaction with the organosilica gels upon curing of matrices, several mixtures of TiO_2_ and the respective matrices at different weight ratios were also analysed by Raman spectroscopy. In the case of ANC, the Raman study of different mixtures of TiO_2_ nanoparticles (nTiO_2_-A) with the respective matrix (m-ANC) did not allow to detect any new signals ascribable to a chemical interaction between the two components. This agrees with the above made hypothesis that nTiO_2_-A particles may be actually surrounded by a shell of adsorbed 1,2-propanediol molecules keeping them protected from direct interaction with the organosilica gel. In the case of WNC, changes in the relative intensities and small shifts of several signals of the matrix were observed in the spectra of mixtures, indicating that this matrix undergoes a possibly conformational and/or structural rearrangement upon addition of TiO_2_ nanoparticles (Figure 4). Furthermore, it was possible to detect a signal at 1013 cm^−1^, which can be only observed in the spectra of 1:1 and 1:5 nTiO_2_-W/m-WNC mixtures, that is, with an excess of silane over TiO_2_. According to the literature [52], this signal could be ascribed to the formation of a covalent Si-O-Ti link between TiO_2_ and the silanol groups resulting from the hydrolysis of an aminoalkylalkoxysilane compound. The chemical interaction between TiO_2_ and the organosilica gel of WNC probably contributes to an increase in the adhesion forces between nanoparticles and the surrounding matrix, thus improving the retention of titania on the treated stone surfaces. This argument will be resumed in the following, while discussing the durability of coatings and, notably, the effects of rain wash-out (Section 3.3).

#### 3.1.3. Absorption of Protective Treatments

The volumes of WNC, ANC and their respective matrices (m-WNC and m-ANC) absorbed by Ajarte and Obernkirchen (Table 4) indicate that ANC/m-ANC saturate the pore volume of both stones to nearly the same extent as water (Figure 2). This is evidence of the fact that they encounter no difficulty in penetrating into the stone capillary networks, in agreement with their non-aggregating behaviour and low viscosity (Table 3). On the other hand, the absorption of WNC/m-WNC turns out to be much more difficult, most likely due to the higher particle size and greater aggregation of their components. Such effect is especially evident on Ajarte, whose lower average pore diameter and higher pore surface area (Table S1) provide a greater interaction between stone and penetrating fluids and pose higher limitations on the mobility of nanoparticles.

Then, the comparison between nanocomposites and their respective matrices shows that, while ANC/m-ANC display the same absorption behaviour, the uptake of WNC is considerably lower than that of m-WNC, all of which is once again consistent with the values of particle size reported in Table 3.

### 3.2. Testing of Treated Lithotypes

#### 3.2.1. Surface Colour Monitoring

Colour measurements (Table 5) indicate that both nanocomposites (WNC and ANC) have good aesthetic compatibility on Ajarte stone, producing colour variations (ΔE*) well below the threshold value of 5 [41], as it had already been assessed in our previous study [36]. On Obernkirchen, WNC still exhibits a good compatibility, while ANC gives rise to a greater chromatic alteration as a result of darkening (ΔL* = −8) and yellowing (Δb* = 5), which are probably associated with a more extensive coverage of the quartz grains by low surface energy alcohol-based products. The colour analysis of stones treated with m-WNC and m-ANC indicates that nanocomposites behave in much the same way as their respective matrices, hence the addition of TiO_2_ nanoparticles does not involve further chromatic variations.

#### 3.2.2. Evaluation of Surface Morphology

A comparison of the surface morphology of untreated and treated stones through scanning electron microscopy was first carried out (see Figures S2 and S3 in Supplementary Materials). In the case of Ajarte stone, which is characterized by a fine-grained porous microstructure of calcite crystals, the natural stone features are almost completely retained after treatment, even though few localized accumulations of the treatments can be detected. As regards WNC, observations in SE mode allow to detect a diffuse bridging of intercrystalline gaps. In the case of Obernkirchen, that is, a stone characterized by medium-coarse size quartz grains embedded in a very fine-grained silicate cement with evident voids and diffuse network of pores, the coatings can be easily detected even from BSE images. A smoothening of the surface morphology, with covering of the clasts and filling of the surface pores, especially in the case of ANC, can be observed.

AFM measurements of surface roughness (Table 6) were performed to investigate at nanoscale the stone/coating interaction and, in particular, to assess how TiO_2_ nanoparticles contribute to the modification of the surface textural properties of stones. The two lithotypes are characterized by different textural features, as Ajarte displays a much greater surface nanoroughness with respect to Obernkirchen. Furthermore, the two alkylalkoxysilane matrices interact with stones in a completely different way: the water-based matrix (m-WNC) reduces surface roughness drastically, as it can be expected from its poorer penetration, more so on Ajarte stone, which exhibits a higher intrinsic nanoroughness. On the other hand, the alcohol-based matrix (m-ANC) tends to enhance the naturally rough topography of stones, notably in the case of Obernkirchen, that is, the stone with lower intrinsic nanoroughness. The addition of titania can be seen to produce different effects depending on the amount of nanoparticles and the properties of the matrix. In ANC, that is, the product with lower titania content (Table 2), the addition of nanoparticles seems to reduce the roughness-inducing effect of the matrix. In the case of WNC, which is characterized by a higher loading of titania and by a matrix with pronounced texture smoothening effects, the presence of nanoparticles can be seen to slightly reduce these effects on Ajarte and results in only a minor increase in surface nanoroughness on Obernkirchen. In conclusion, while the interaction of alkylalkoxysilane matrices with stones is clearly modified by the addition of nanoparticles, there is no clear evidence that the latter contribute to enhancing surface roughness. The lack of this effect is probably due to the comparatively small amount of titania in the two formulations, which is much lower than applied in one previous study where a contribution of nanoparticles to surface roughness was clearly observed [19]. Besides that, it must be considered that on porous stones such contribution is further reduced by the penetration of nanoparticles into the porous matrix.

#### 3.2.3. Evaluation of Water Absorption and Surface Wettability

Our previous studies regarding the protective performance of WNC and ANC [36,37] showed that these products are able to effectively reduce the water uptake both at short-term (AC) and long-term (CI_rel_) contact, with a slightly higher effectiveness of the alcohol-based product ANC, as can be expected from its higher penetration (Table 4). The results of water absorption measurements by capillarity performed in this study (Table 7) show clearly that nanocomposites (WNC, ANC) and corresponding TiO_2_-free matrices (m-WNC, m-ANC) behave in a very similar way, thus proving that the addition of nanoparticles, though reducing the penetration of WNC into the stone pores (Table 4), does not compromise the effectiveness of matrices in protecting stones from water capillary absorption. 

This is a rather interesting result, which suggests that the homogeneity of surface deposition and pore hydrophobization are more critical factors in determining a satisfactory water-barrier effect than is the total amount of applied product. Among the two stones, Ajarte turns out to be harder to protect, which is expected on the basis of its high number of small-size pores (Figure S1) that treatments are less likely to enter and protect. 

Both nanocomposites and corresponding matrices are also able to impart high water-repellency (Table 8) to the stone surfaces on which they are applied, yielding contact angles higher than 130°, which amount to so-called “superhydrophobic” behaviour [53]. With the exception of WNC, all treatments give slightly higher contact angles on Ajarte than Obernkirchen, which is consistent with the greater amounts of products absorbed by this stone and its naturally higher surface nanoroughness (Table 6) contributing to water-repellency via the Cassie-Baxter state. As pointed out in the discussion of AFM results (Section 3.2.2), the comparison between nanocomposites and matrices shows that the presence of TiO_2_ nanoparticles does not contribute to the water-repellency of matrices through an increase in surface nanoroughness. Actually, it can be observed that ANC gives rise to slightly lower contact angles with respect to its matrix, which can be reasonably ascribed to the higher surface roughness induced by the latter (Table 6). As regards WNC, the nanocomposite behaves in much the same way as m-WNC on Obernkirchen, whereas on Ajarte it induces a contact angle about 8° lower than that provided by the matrix. In this case, the difference is probably due to a less effective surface coverage of one of the three specimens used for the test (as the high standard deviation of measurements indicates), again suggesting that, on a stone characterized by a large number of very small intercrystalline pores, the product is somewhat less effective than its matrix in providing a uniform hydrophobization of the pores’ walls due to the larger size of its aggregates (Table 3).

#### 3.2.4. Evaluation of Photocatalytic Activity

In a previous study evaluating the photocatalytic properties of WNC and ANC on Ajarte stone [36], it was shown that specimens treated with WNC exhibit a considerably faster colourant degradation with respect to a non-photocatalytic reference product, thus proving that TiO_2_ nanoparticles present in the formulation have a well-defined photocatalytic action. The trend of colourant degradation during the exposure to Xenon lamp irradiation also indicated that the kinetics of photocatalyzed discolouration is very fast within the first 30 min of irradiation and already attains a plateau at 90 min, after which the rate is gradually diminished due to the parallel progress of the slower non-catalysed photo-oxidative reaction. In the case of ANC, a less relevant increase in the discolouration rate was observed, pointing out a lower yet still visible photocatalytic activity. In the present research, analogous results for WNC and ANC are also achieved on Obernkirchen (Table 9), in spite of the different microstructure and mineralogical composition of this stone. 

Furthermore, the comparison between nanocomposites and matrices allows to make a more accurate evaluation of the role of TiO_2_ nanoparticles in the different rates of discolouration displayed by the two nanocomposites. The higher photocatalytic activity of WNC has been explained by its higher titania content (Table 2) and by the observed tendency of TiO_2_ nanoparticles in ANC to aggregate during the curing of the product, thus reducing their specific surface area [36]. The ‘hybrid’ character of these nanoparticles, which are probably surrounded by a shell of chemisorbed 1,2-propanediol molecules (as discussed in Section 3.1.2), may also contribute to the reduction of their photoactivity.

### 3.3. Evaluation of the Durability of Protective Treatments

The chemical stability of nanocomposites and TiO_2_-free matrices upon UV light irradiation was studied by referring to their FTIR spectra, recorded before and after the UV ageing procedure and to the modification of capillary water absorption of stone specimens.

**Figure 5:**
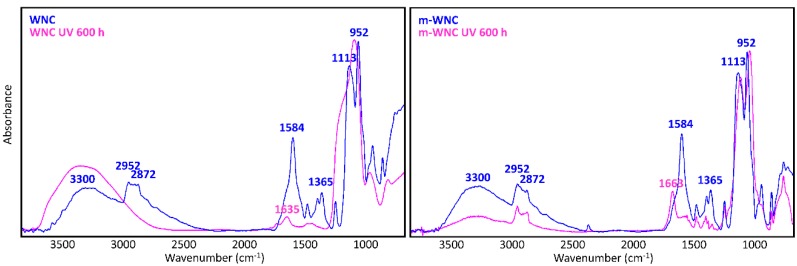
μ-FTIR spectra (ATR mode) of WNC and m-WNC films on glass slides *before* and *after* UV ageing for 600 h.

**Figure 6:**
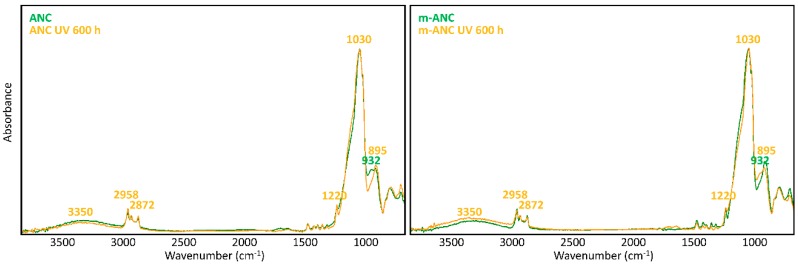
μ-FTIR spectra (ATR mode) of ANC and m-ANC films on glass slides *before* and *after* UV ageing for 600 h.

A glance at Figure 5 and Figure 6 shows immediately that WNC/m-WNC have a markedly different behaviour with respect to ANC/m-ANC. While in the first case (Figure 5) spectra before and after ageing show clear differences, which become very impressive in the presence of TiO_2_ nanoparticles, the spectra of ANC and m-ANC (Figure 6) after irradiation are practically superimposable to those of the unaged materials. It is worth noticing that in the ANC/m-ANC case, the IR spectrum is quite simple, showing strong bands in the O-H and C-H stretching regions (3500–2800 cm^−1^), weak features in the 1500–1300 cm^−1^ region and a dominant absorption in the region of Si-O stretching modes (maximum at 1030 cm^−1^). On the contrary, the spectra of WNC/m-WNC (Figure 5) are characterized by the occurrence of many very strong absorption features, which can be ascribed to the presence of species containing polar groups. In particular, features due to the amine groups are expected based on the material formulation (Table 1). As it will be better analysed in the following discussion, these chemical groups undergo chemical transformations upon irradiation, especially in presence of TiO_2_ nanoparticles. In the case of m-WNC, the observed changes of the spectral pattern upon irradiation cannot be ascribed to photo-oxidative degradation, pointing out a rather good chemical stability. In particular, the retention of the C-H stretching band (peaks at 2952–2872 cm^−1^) and the one due to Si-C stretching at 1230 cm^−1^ indicate that the alkyl moiety of the matrix has not been compromised. The broad feature with maximum at 3330 cm^−1^ can be ascribed to contributions from O-H and N-H stretching modes: Its reduction in intensity can be related to the heat-induced evaporation of water molecules trapped inside the gel matrix, as well as to reactions involving amine groups. For this reason, it is impossible to say whether oxidation products containing hydroxyl groups are formed upon ageing. Other relevant changes in the spectrum are the vanishing of the peak at 1584 cm^−1^ (this feature is compatible with the N-H bending of the amine group, involved in hydrogen bonding [54]), with parallel appearance of one at 1663 cm^−1^. This last feature could be ascribed to the presence of amide groups, whose formation is consistent with the reactivity displayed by some aminoalkylalkoxysilanes towards carbon dioxide [55]. Actually, by considering that the strong band of the unaged sample at 1584 cm^−1^ shows a shoulder at about 1660 cm^−1^, it is possible that this peak at 1663 cm^−1^ is already present before irradiation. Quite different from its pure matrix, the ageing of WNC, where TiO_2_ is present, leads to the complete mineralization of the organic counterpart and rearrangement of the silica-gel network, suggested by the increase of the O-H stretching band around 3300 cm^−1^, the appearance of a distinct signal at 1635 cm^−1^ due to O-H bending, the disappearance of the C-H stretching peaks (2872–2952 cm^−1^) and the change in the shape of the Si-O-Si stretching band around 1110 cm^−1^. A decrease in the intensity of the TiO_2_-related band below 700 cm^−1^ can also be observed, yet the form of the band is retained, indicating that TiO_2_ is still present in the composite. Therefore, the addition of TiO_2_ nanoparticles at nearly 1% *w*/*w* leads to a drastic modification of the ageing resistance of the matrix, accelerating photo-oxidative reactions with consequent predictable loss of its water-repellent properties. The IR analysis of ANC and its matrix upon ageing (Figure 6) shows instead in both cases a complete retention of the original spectral features (with the only exception of a small peak appearing at 932 cm^−1^, probably due to some modification occurring in the siloxane backbone). In particular, no decrease of C-H related bands and no increase of those related to –OH groups can be observed, hence a photo-oxidative degradation of the alkyl chains responsible for coating’s hydrophobicity, either in the pure matrix or in the nanocomposite, can be ruled out. Therefore, in this case, the addition of a lower amount of TiO_2_, possibly in a less photo-active form (see Section 3.1.2), does not seem to compromise the chemical stability of the matrix.

**Figure 7:**
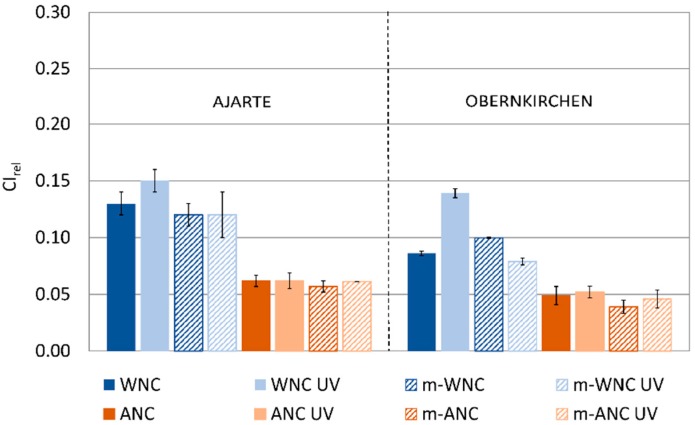
CI_rel_ values of Ajarte and Obernkirchen stones treated with WNC, ANC, m-WNC and m-ANC *before* and *after* UV ageing for 600 h.

The trends of change in capillary water absorption for stone specimens treated with WNC, ANC and their respective matrices after UV ageing (Figure 7) are in agreement with the findings of IR analysis. Indeed, while TiO_2_-free matrices look unaffected by UV light irradiation and retain their good protective performance, the addition of TiO_2_ nanoparticles in the products leads to different effects, from no increase in water absorption in the case of ANC to a moderate increase in the case of WNC, that is, the product with higher titania content. 

On one hand, this confirms that the presence of a higher loading of TiO_2_ nanoparticles, contributing to the UV-induced degradation of the hydrophobic moiety of the matrix, ends up reducing the “protective ability” of the coating towards the capillary absorption of liquid water. However, from the viewpoint of coating performance, this reduction of protective ability is quite limited, while the fact that the water-barrier effect is not severely compromised proves that the degradative effects do not extend beyond a small depth from the stone surface, thus leaving the bulk of the coating inside the pores largely unaffected. This is a rather significant result, which adds an important piece of information to the knowledge of the durability of photocatalytic coatings applied on stones.

**Figure 8:**
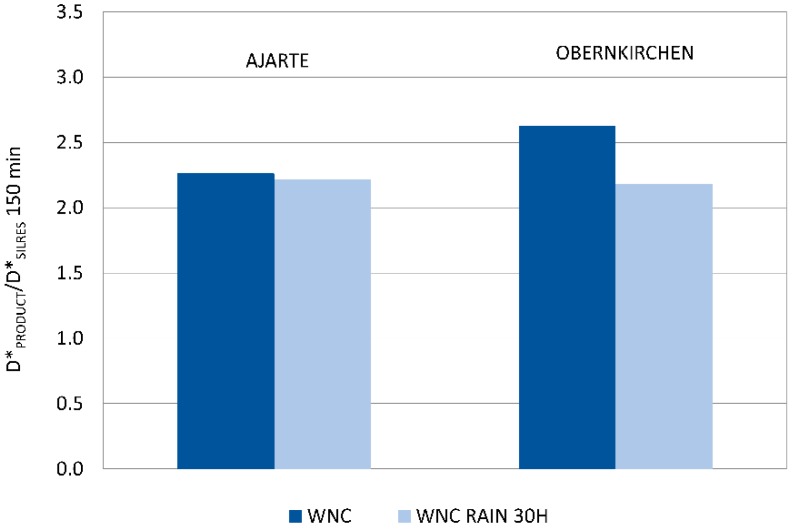
Ratio of discolouration values (D*) of WNC (D*_PRODUCT_) and corresponding values for the reference non-photocatalytic product Silres BS 290 (D*_SILRES_) at 150 min irradiation for Ajarte and Obernkirchen stones after rain ageing for 30 h.

A second, not less important result, comes from the assessment of the mechanic stability of TiO_2_ nanoparticles towards rain wash-out, which was only performed on specimens treated with WNC, that is, the product with distinct photocatalytic features (Section 3.2.4). The results of the Rhodamine test performed after ageing (Figure 8) show that a good level of photocatalytic activity is retained on both Ajarte and Obernkirchen stones, indicating the persistence of TiO_2_ nanoparticles on the stone surfaces. This is also attested by EDX analysis showing the presence and homogeneous distribution of silicon and titanium. Poor adhesion to stone surfaces is notoriously one of the drawbacks of using bare TiO_2_ dispersions and one of the reasons for switching over to nanocomposite formulations. The assessed “physical-mechanical” stability of TiO_2_ nanoparticles in WNC is an argument in support of the claim that a good adhesion of TiO_2_ to stone is achieved through the embedding organosilica gel matrix. The organosilica gel of WNC, which is based on an aminoalkylalkoxysilane precursor (Table 1), is expected to have good adhesion properties towards both calcareous and siliceous stones. Besides that, as the interpretation of Raman spectra suggested (Section 3.1.2.), a good interaction is achieved between TiO_2_ nanoparticles and the matrix, probably resulting in a greater stability of the former inside the protective coating.

## 4. Conclusions

This study considered the role of TiO_2_ nanoparticles in the stone/coating interaction and the durability of two photocatalytic nanocomposites for the protection of stones, which rely on the combination of alkylalkoxysilane matrices (water- or alcohol-based) with different TiO_2_ nanoparticles. The specific aims of the research were to assess how TiO_2_ nanoparticles interact with matrices and how they modify the latters’ interaction with stones and the durability of the coatings. To address these aims, the two nanocomposite formulations were compared to the respective TiO_2_-free matrices and their protective behaviour was assessed on two porous stones with different microstructural and mineralogical properties. The following results were achieved:

- The aggregate size and reactivity of the nanocomposite formulations and the mean pore diameter of stones turned out to be the most relevant factors determining their different absorption and penetration. The addition of TiO_2_ nanoparticles to alkylalkoxysilane matrices was shown to produce different effects depending on the reactivity of the matrix and the amount of nanoparticles. For the less reactive alcohol-based matrix (m-ANC), the nanocomposite retains the penetration ability and the protective properties of the silane precursor. For the more reactive water-based matrix (m-WNC) an effect on the aggregation state of the alkylalkoxysilane component was observed, resulting in a lower penetration of the composite product.
- Despite its lower penetration, the water-based nanocomposite WNC showed a good protective performance, particularly on the stone with higher mean pore diameter (Obernkirchen), indicating that the homogeneity of surface deposition and pore hydrophobization are more critical factors in determining a good water-repellency than is the total amount of applied product. The addition of nanoTiO_2_ to m-WNC did not modify the protective effectiveness of the matrix, whilst it gave rise to the desired significant photocatalytic activity. In the case of the alcohol-based nanocomposite ANC, in spite of the good penetration of the treatment and very good protective performance, the reduced amount of TiO_2_ nanoparticles did not allow to obtain distinct photocatalytic features. Moreover, this product caused a visible chromatic alteration on the siliceous stone (Obernkirchen) and in real conditions it is highly preferred to work with water-based formulations.
- The investigation of the durability of coatings upon exposure to UV light clarified that TiO_2_ nanoparticles, at the higher concentration found in WNC, contribute to a photo-induced oxidative degradation of the organic component of the matrix, revealed by FTIR analysis. Nevertheless, an important result of this study was that this degradation does not substantially compromise the effectiveness of the coating in reducing water capillary absorption, because it does not extend to the pore network under the surface where the coating imparts most of its water-barrier effect. Furthermore, the good interaction achieved between TiO_2_ nanoparticles and the embedding matrix in WNC, assessed through Raman analysis, resulted in a stable anchoring of nanoparticles to the stone surfaces even after prolonged exposure to in-lab simulated rain wash-out.

As a complementary part to this study, the nanocomposite coatings have also been applied in situ on small pilot areas of the Cathedrals of Vitoria-Gasteiz and Cologne, where the selected stones are used as building materials and a long-term on-site monitoring of their performance is currently underway in order to examine their durability under real environmental conditions.

## Figures and Tables

**Table 1:** Main properties of the components used for the preparation of WNC and ANC, namely TiO_2_ NPs (nTiO_2_-W and nTiO_2_-A) and alkylalkoxysilane matrices (m-WNC and m-ANC), as reported by the producers: chemical composition, solvent, concentration (*w*/*w*) and nanoparticle size (nm).

Component	Description	Solvent	Concentration	NPs Size ^1^
nTiO_2_-W	TiO_2_ NPs	water (pH 1.5)	5.5%	50 ± 10
nTiO_2_-A	TiO_2_ NPs	1,2-propanediol	12%	20 ± 5
m-WNC	*n*-propyl trimethoxysilane tris(propyltrimethoxysilyl)amine formic acid	water (pH 4.5)	15%	-
m-ANC	2-methylpropyl trimethoxysilane ethyl orthosilicate butyl orthotitanate (cat.)	2-propanol	40%	-

^1^ measured by Dynamic Light Scattering (DLS).

**Table 2:** Composition (*w*/*w*) of WNC and ANC, as reported by the producers.

Product	Solvent	Composition
WNC	water (pH 4.5)	0.96% nTiO_2_-W 15% m-WNC
ANC	2-propanol	0.12% nTiO_2_-A 40% m-ANC

**Table 3:** Main properties of the products (WNC, ANC) and respective matrices (m-WNC and m-ANC): density (g/cm^3^), viscosity (mPa·s) and particle size (nm).

	Density	Viscosity	Particle Size
WNC	1.03	10 ± 1	105.9 ± 0.4 ^1^
m-WNC	1.03	10 ± 1	82.8 ± 0.2
ANC	0.84	7 ± 1	25 ± 1 ^1^
m-ANC	0.84	11 ± 3	-

^1^ data taken from [37].

**Table 4:** Volume of liquid treatments (μL/cm^2^) absorbed by Ajarte and Obernkirchen stones. Values are averaged on 3 specimens.

	WNC	m-WNC	ANC	m-ANC
Ajarte	122 ± 17	203 ± 47	408 ± 6	411 ± 7
Obernkirchen	74 ± 5	213 ± 1	236 ± 19	219 ± 5

**Table 5:** Values of ΔE*, ΔL*, Δa* and Δb* of Ajarte and Obernkirchen stones treated with WNC, ANC and their matrices. Values are averaged on 3 specimens.

		ΔE*	ΔL*	Δa*	Δb*
AJARTE	WNC	1.5 ± 0.3	−0.9 ± 0.3	−0.36 ± 0.06	1.1 ± 0.3
	m-WNC	1 ± 1	−0.6 ± 0.7	−0.3 ± 0.1	1.1 ± 0.9
	ANC	2.2 ± 0.6	−2.0 ± 0.8	0.21 ± 0.09	−0.3 ± 0.9
	m-ANC	4 ± 2	−3 ± 1	0.3 ± 0.3	1.5 ± 0.6
OBERN.	WNC	2.6 ± 0.6	−2.3 ± 0.5	0.15 ± 0.08	1.1 ± 0.4
	m-WNC	2.5 ± 0.9	−1.7 ± 0.7	0.08 ± 0.09	1.8 ± 0.5
	ANC	10 ± 1	−8 ± 1	1.3 ± 0.3	5 ± 1
	m-ANC	9 ± 1	−7 ± 2	1.0 ± 0.2	4.7 ± 0.6

**Table 6:** Values of root mean square (RMS) surface Roughness (nm) of Ajarte and Obernkirchen stones: untreated and treated with WNC, m-WNC, ANC and m-ANC.

	Untreated	WNC	m-WNC	ANC	m-ANC
Ajarte	33 ± 3	5	1	37	38
Obernkirchen	7 ± 3	12	3	3	39

**Table 7:** Amount of water absorbed per unit area at 96 h (Q_i_, mg·cm^−2^) and absorption rate at 30 min (AC, mg·cm^−2^·s^−1/2^) *before* (nt) and *after* (t) treatment with WNC, m-WNC, ANC and m-ANC for Ajarte and Obernkirchen stones and respective values of Relative Capillary Index (CI_rel_). Values are averaged on 3 specimens.

		Q_i_ nt	Q_i_ t	AC nt	AC t	CI_rel_
AJARTE	WNC	430 ± 20	79 ± 6	4.2 ± 0.6	0.144 ± 0.007	0.132 ± 0.008
	m-WNC	430 ± 30	79 ± 3	5.2 ± 0.9	0.132 ± 0.001	0.12 ± 0.01
	ANC	447 ± 3	36 ± 3	4.6 ± 0.8	0.112 ± 0.004	0.063 ± 0.005
	m-ANC	438 ± 2	34 ± 4	5.1 ± 0.2	0.106 ± 0.003	0.061 ± 0.008
OBERN.	WNC	257 ± 6	33 ± 5	2.8 ± 0.2	0.076 ± 0.005	0.09 ± 0.01
	m-WNC	250 ± 4	36.4 ± 0.7	3.4 ± 0.4	0.070 ± 0.004	0.099 ± 0.001
	ANC	260 ± 20	18 ± 4	3.7 ± 0.6	0.075 ± 0.006	0.052 ± 0.008
	m-ANC	240 ± 10	14 ± 5	3.2 ± 0.6	0.057 ± 0.005	0.05 ± 0.01

**Table 8:** Values of static contact angle (θ, °) of water *before* (nt) and *after* (t) treatment with WNC, m-WNC, ANC and m-ANC for Ajarte and Obernkirchen stones. Values for treated stones are averaged on 2 specimens.

		θ nt	θ t
AJARTE	WNC	<10 ^1^	131 ± 14
	m-WNC		139 ± 3
	ANC		138 ± 2
	m-ANC		142 ± 4
OBERN.	WNC	21 ± 2	140 ± 2
	m-WNC		138 ± 1
	ANC		133 ± 1
	m-ANC		137 ± 1

^1^ contact angles on Ajarte are too low to be measured.

**Table 9:** Ratio of discolouration values (D*) of Ajarte and Obernkirchen stones treated with WNC/ANC (D*_PRODUCT_) and corresponding values for the reference non-photocatalytic product Silres BS 290 (D*_SILRES_) after 30, 90 and 150 min irradiation.

		D*_PRODUCT_/D*_SILRES_		
		30 min	90 min	150 min
AJARTE	WNC ^1^	5.6	3.9	3.3
	m-WNC	0.7	0.6	0.6
	ANC ^1^	0.2	2.0	2.0
	m-ANC	0.7	1.0	0.8
OBERN.	WNC	4.7	5.2	3.7
	m-WNC	0.8	1.1	0.5
	ANC	2.1	2.2	1.7
	m-ANC	1.1	-	0.7

^1^ data taken from [36].

## Supplementary Materials

The following are available online at http://www.mdpi.com/1996-1944/11/11/2289/s1. Table S1: Microstructural features of lithotypes: total open porosity (vol%), average pore diameter (μm), pore surface area (m^2^/g) and bulk density (g/cm^3^); Figure S1: Pore-size distribution of lithotypes; Figure S2: SEM images of Ajarte stone in BSE (left) and SE (right) mode: Untreated (A) and treated with WNC (B), m-WNC (C), ANC (D), m-ANC (E); Figure S3: SEM images of Obernkirchen stone in BSE (left) and SE (right) mode: Untreated (A) and treated with WNC (B), m-WNC (C), ANC (D), m-ANC (E).
