# Supplementary material for: Stone/Coating Interaction and Durability of Si-Based Photocatalytic Nanocomposites Applied to Porous Lithotypes
Source: Materials (Basel). 2018 Nov 15;11(11):2289. doi: 10.3390/ma11112289 (PMC6266438; doi:10.3390/ma11112289)
Supplement: Supplementary file 1 [file materials-11-02289-s001.pdf]

materials

MDPI

Supplementary materials

Stone/coating interaction and durability of Si-based photocatalytic nanocomposites applied to porous lithotypes

Marco Roveri [1,*], Francesca Gherardi [2], Luigi Brambilla [1], Chiara Castiglioni [1] and Lucia Toniolo [1]

[1] Politecnico di Milano, Dipartimento di Chimica, Materiali e Ingegneria Chimica "G. Natta", Milano, Italy;

[2] University of Lincoln, School of Chemistry, Lincoln, UK;

* Correspondence: marco.roveri@polimi.it; Tel.: +39-02-2399-3143

Received: date; Accepted: date; Published: date

Table S1. Microstructural features of lithotypes: total open porosity (vol%), average pore diameter (μm), pore surface area (m^2/g) and bulk density (g/cm^3). Data obtained from Mercury Intrusion Porosimetry measurements on 2 specimens per lithotype.

	Open porosity	Av. pore diameter	Pore surface area	Bulk density
Ajarte	23.5±0.4	0.17±0.03	2.7±0.5	2.08±0.01
Obernkirchen	24.1±0.1	0.8±0.3	0.7±0.3	2.08±0.06

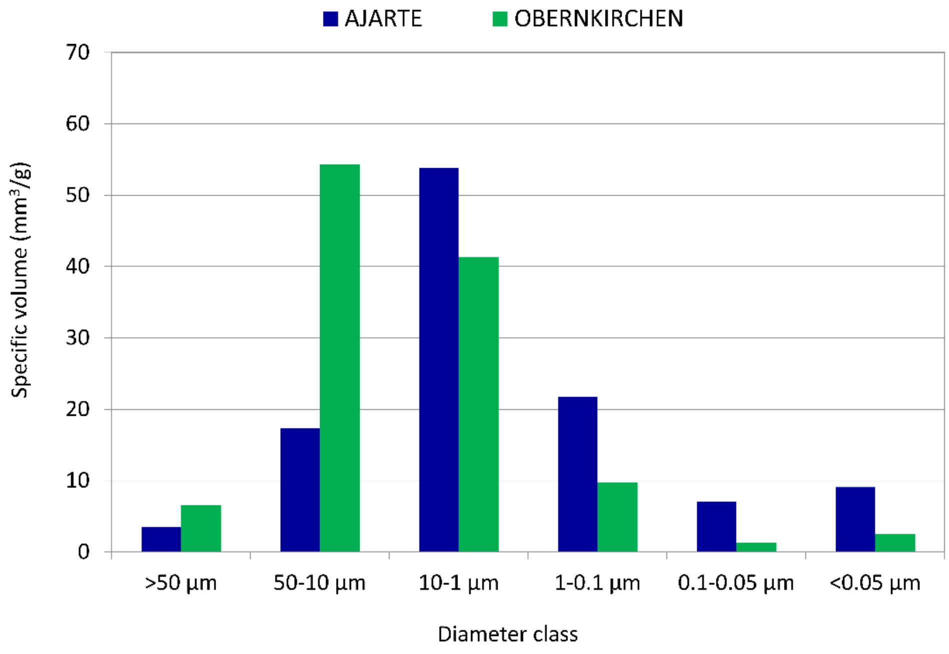

Figure S1. Pore-size distribution of lithotypes (from Mercury Intrusion Porosimetry measurements).

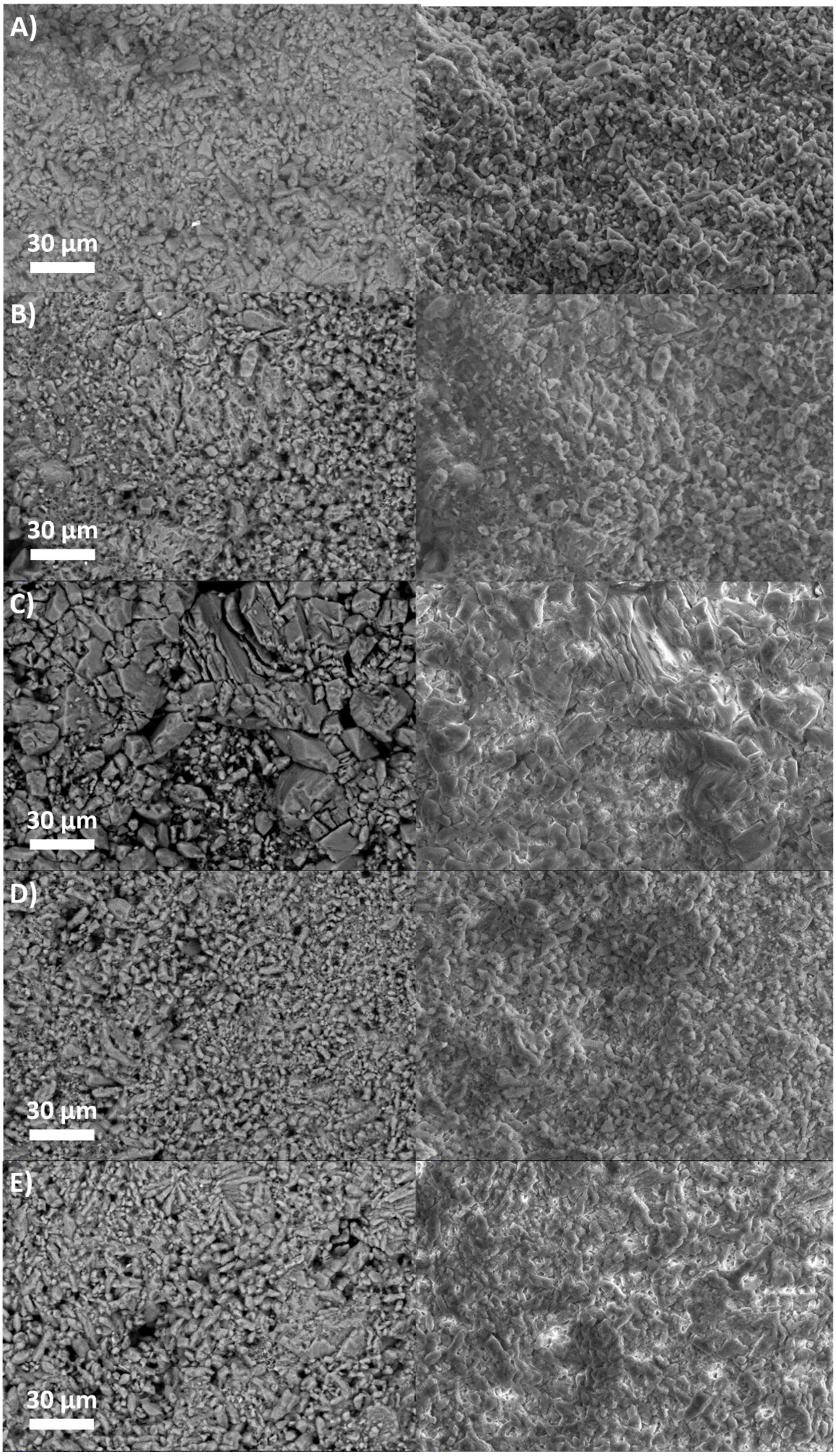

452 **Figure S2**. SEM images of Ajarte stone in BSE (left) and SE (right) mode: *untreated* (**A**) and *treated* with
453 WNC (**B**), m-WNC (**C**), ANC (**D**), m-ANC (**E**).

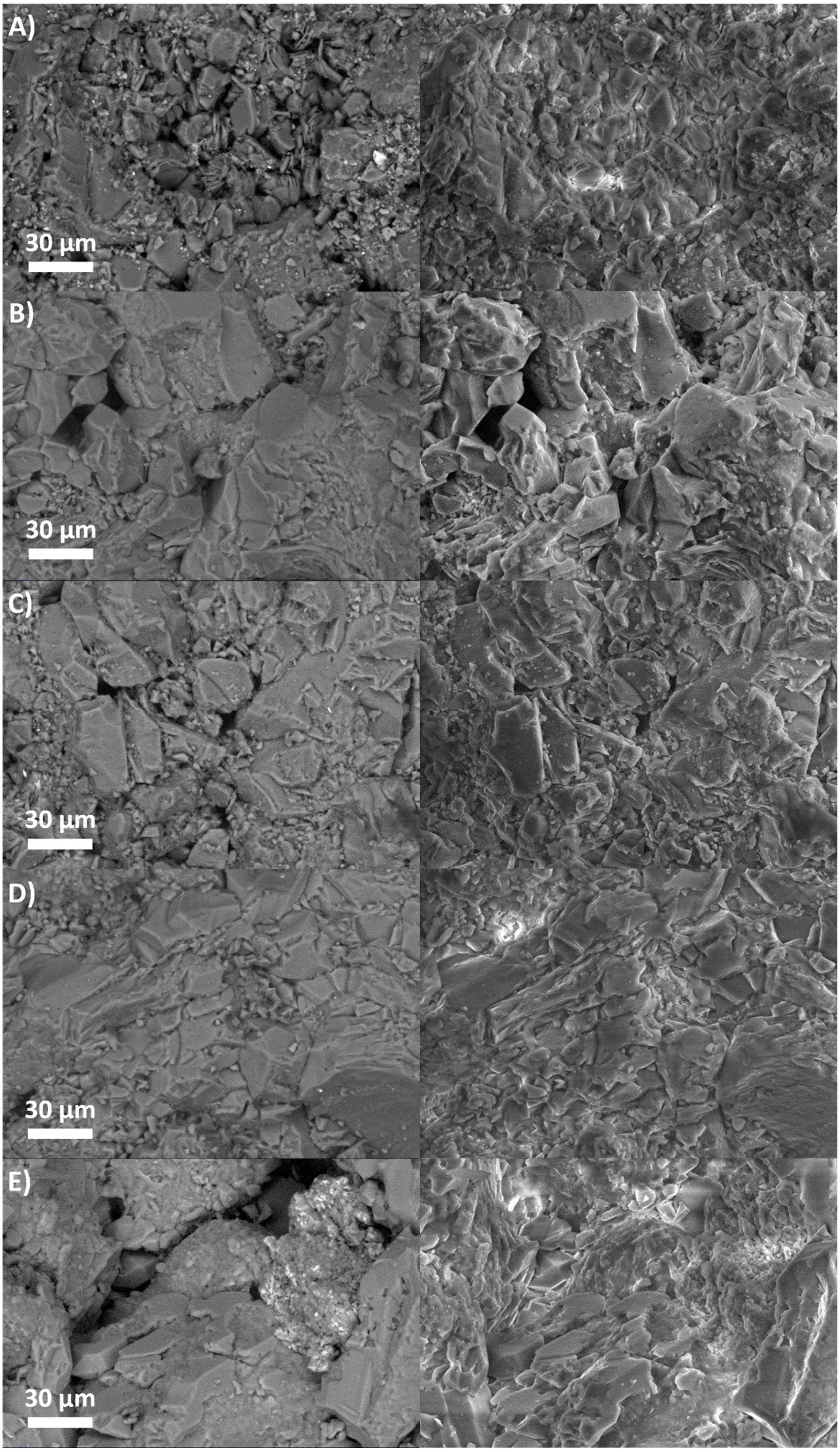

Figure S3. SEM images of Obernkirchen stone in BSE (left) and SE (right) mode: untreated (**A**) and treated with WNC (**B**), m-WNC (**C**), ANC (**D**), m-ANC (**E**).

materials

460
